# Supplementary material for: Long-term wage inequality in imperial China: From 202 BCE to 1912 CE
Source: PLoS One. 2025 Jan 24;20(1):e0315627. doi: 10.1371/journal.pone.0315627 (PMC11761105; doi:10.1371/journal.pone.0315627)
Supplement: Attachment — Submitted filename: Review Report.docx [file pone.0315627.s002.docx]

This study compiles historical records to benchmark wage inequality in imperial China from 202 BCE to 1912 CE. The authors find a gradual increase in wage inequality from the Han to the mid-Tang dynasty and a decline from the late-Tang to the Qing dynasty, presenting an overall inverted U-shaped pattern, nested with short-term “inverted u” cycles. Contrarily, wage inequality among officials exhibited a secular decline over two millennia. The paper also proposes a unified theoretical framework to explain inequality evolution in pre-industrial society.

The topic of this paper is intriguing. The extensive historical span and comprehensive data analysis provide valuable insights into the long-term wage inequality in imperial China. I recommend the editor to consider a quick minor revision for publication. Here are three minor comments to enhance the manuscript.

Comment 1: The discussion on social mobility would benefit from distinguishing between intergenerational and intragenerational mobility. For example, the “Ke Ju” system might have promoted intergenerational mobility by allowing young individuals from lower-class families to ascend social ranks through the examination system, thereby reducing inequality. This aligns with the observed trend of decreasing inequality after the peak during the Sui-Tang period. Conversely, the evolution of social norms beginning in the Song dynasty may have restricted rights, potentially reducing intragenerational mobility. This observation corresponds with Yang & Zhou’s (2022) findings on the positive correlation between inequality and intragenerational mobility (Gatesby curve).

Comment 2: On page 10, the manuscript states, “We use equivalent volumes of rice to measure the real wage.” However, rice yields would have increased with agricultural advancements, potentially affecting the measurement of both peasants’ income and officials’ land rent income. The authors should provide additional clarification, such as explaining that agricultural output is distributed between officials and farmers through land rent, and therefore, the increase in unit land yield affects both groups proportionally. Furthermore, discussing the impact of rising land rent (relative share r/(1-r)) on the distribution of agricultural output between the two classes could be beneficial.

Comment 3: The paper measures inter-class wage inequality using “the wage ratio between officials and peasants”. As commerce and industry developed since the Tang dynasty, how might wage inequality measured the relative wage indicator based on peasant income be influenced? A brief discussion on this point would be helpful.

I look forward to seeing the revised version of this insightful manuscript.

Best regards,

Xiaoliang
