# Supplementary material for: Long-term wage inequality in imperial China: From 202 BCE to 1912 CE
Source: PLoS One. 2025 Jan 24;20(1):e0315627. doi: 10.1371/journal.pone.0315627 (PMC11761105; doi:10.1371/journal.pone.0315627)
Supplement: Attachment — Submitted filename: Response to Reviewers.docx [file pone.0315627.s003.docx]

**Rebuttal Letter**

Dear Editor and Reviewers,

Thank you very much for the precious opportunity to improve our paper based on your insightful, constructive comments. To reflect our work and improvements, we provide a point-to-point response below to the editor’s and reviewers’ reports. Thanks again.

| **Comments from the Editor** | **Responses** |
| --- | --- |
| 1. Please ensure that your manuscript meets PLOS ONE's style requirements, including those for file naming. | We have now revised the style according to the requirements. |
| 2. Thank you for stating the following financial disclosure: This study was supported by the General Project of the National Social Science Fund of China (Grant Number: 23BJY133).  Please state what role the funders took in the study. If the funders had no role, please state: "The funders had no role in study design, data collection and analysis, decision to publish, or preparation of the manuscript." | We have added "The funders had no role in study design, data collection and analysis, decision to publish, or preparation of the manuscript." in the financial disclosure.  This study was supported by the General Project of the National Social Science Fund of China (Grant Number: 23BJY133). The funders had no role in study design, data collection and analysis, decision to publish, or preparation of the manuscript. |
| 3. Please ensure that you refer to Figure 7 in your text as, if accepted, production will need this reference to link the reader to the figure. | The original Figure 7 (now Figure 8) is cited in the text right before it. |
| 4. Please include captions for your Supporting Information files at the end of your manuscript, and update any in-text citations to match accordingly. Please see our Supporting Information guidelines for more information. | After careful consideration, we have decided to integrate the table and figure into the supporting information into the main text. Therefore, we no longer have supporting information. |
| 5. Please review your reference list to ensure that it is complete and correct. If you have cited papers that have been retracted, please include the rationale for doing so in the manuscript text, or remove these references and replace them with relevant current references. Any changes to the reference list should be mentioned in the rebuttal letter that accompanies your revised manuscript. If you need to cite a retracted article, indicate the article’s retracted status in the References list and also include a citation and full reference for the retraction notice. | We have carefully checked all references and updated them according to the requirements. |

| **Comments from Reviewer #1** | **Responses** |
| --- | --- |
| Comment 1: The discussion on social mobility would benefit from distinguishing between intergenerational and intragenerational mobility. For example, the “Ke Ju” system might have promoted intergenerational mobility by allowing young individuals from lower-class families to ascend social ranks through the examination system, thereby reducing inequality. This aligns with the observed trend of decreasing inequality after the peak during the Sui-Tang period. Conversely, the evolution of social norms beginning in the Song dynasty may have restricted rights, potentially reducing intragenerational mobility. This observation corresponds with Yang & Zhou’s (2022) findings on the positive correlation between inequality and intragenerational mobility (Gatesby curve). | Thank you for this constructive suggestion. To reflect this insight, we have added a sentence towards the end of the discussion on social mobility:  While the “Ke Ju” system improved intergenerational mobility, the evolution of social norms beginning in the Song dynasty may have restricted intragenerational mobility, as the rights of individuals were curtailed. This trend aligns with the findings of Yang & Zhou (2022), who suggest that increasing inequality in this period was positively correlated with reduced intragenerational mobility (the Great Gatsby curve). |
| Comment 2: On page 10, the manuscript states, “We use equivalent volumes of rice to measure the real wage.” However, rice yields would have increased with agricultural advancements, potentially affecting the measurement of both peasants’ income and officials’ land rent income. The authors should provide additional clarification, such as explaining that agricultural output is distributed between officials and farmers through land rent, and therefore, the increase in unit land yield affects both groups proportionally. Furthermore, discussing the impact of rising land rent (relative share r/(1-r)) on the distribution of agricultural output between the two classes could be beneficial. | This is a good point. We have added some clarifications based on your suggestion.  However, it is important to note that rice yields would have increased over time due to agricultural advancements, which could potentially affect the measurement of both peasants’ income and officials’ land rent income. To address this, it is essential to clarify that agricultural output was typically distributed between officials and farmers through land rent. As unit land yields increased, both groups would have been affected proportionally since land rent captured a fixed share of the total agricultural output. |
| Comment 3: The paper measures inter-class wage inequality using “the wage ratio between officials and peasants”. As commerce and industry developed since the Tang dynasty, how might wage inequality measured the relative wage indicator based on peasant income be influenced? A brief discussion on this point would be helpful. | This is a good point. We have added a discussion of the limitation of our measure at the end of the conclusion section.  In addition, we use the wage ratio between officials and peasants to measure inter-class wage inequality. However, it is important to consider that as commerce and industry developed, particularly from the Tang dynasty onwards, the economic structure became more diversified, and the relevance of peasant income as a baseline for measuring overall wage inequality may have shifted. The growth of urban centers and the expansion of trade and industrial activities created new sources of wealth and income beyond agriculture. As a result, merchants, artisans, and industrial workers increasingly contributed to the economy, potentially altering the dynamics of income distribution. A more comprehensive measure of wage inequality would need to consider the emerging wage disparities within commerce and industry, as well as the interactions between these sectors and traditional agriculture. |

| **Comments from Reviewer #2** | **Responses** |
| --- | --- |
| 1. Clarify Definitions Early On: The paper discusses terms like "wage inequality" and "intra-class" vs. "inter-class" inequality but could benefit from clearer definitions of these terms early in the paper. A brief explanation of how these concepts are measured (e.g., salary ratios, Gini coefficients) right in the introduction would improve clarity for readers unfamiliar with the technical jargon. | Good point. We have now added the clarification/definition of “intra-class” and “inter-class” inequality in the contribution part of the introduction. |
| 2. Clarify the Role of Social Norms: The paper mentions Neo-Confucianism and other social norms as factors that reduced inequality, but this discussion could be further elaborated. Specifically, the mechanisms through which these social norms influenced wage structures could be clarified and backed by additional historical sources or examples. This would help to strengthen the theoretical argument and provide clearer connections between social norms and economic outcomes. | To reflect your suggestion, we have added an additional paragraph to elaborate how social norms (e.g., Neo-Confucianism) affect inequality.  It emphasized moral integrity, social hierarchy, and the importance of maintaining social harmony. These values reinforced the idea of meritocratic governance through the “*Ke Ju*” examination system, which promoted the rise of individuals based on knowledge and virtue rather than inherited wealth or status. Improved social mobility then induced competition among officials, which further strengthened the emperor’s authority over officials in the *Ming* and *Qing* dynasties. As a result, official wage and wage inequality underwent a secular decline as the bargaining power of the *Shi* class diminished. |
| 3. Include Practical Implications: While the historical context is well-explained, the paper could briefly mention what the findings mean for modern-day inequality studies. A short section or paragraph discussing how this historical analysis could inform current policymaking or research on inequality would provide more relevance to contemporary readers. | This is a good suggestion, which improves the generalizability of our research from imperial China to modern world. We have added a separate paragraph to show how the TIPS framework can apply to the consequences of AI and automation. We also discuss the policy implications for the present day.  These factors are still relevant today. For example, the rapid development and deployment of automation and AI in the past few decades (T) have transformed industries such as manufacturing, retail, and services. While these technologies have increased productivity and efficiency, they have disproportionately benefited highly skilled workers in tech and innovation sectors, while displacing lower-skilled workers in more traditional jobs. This has led to a widening wage gap between highly paid professionals and lower-wage workers, increasing overall wage inequality. In response to the growing inequality caused by technological disruption, governments and institutions (I) have implemented various policies, such as tax incentives for tech companies, deregulation of certain industries, and targeted education and reskilling programs. However, these measures have struggled to keep pace with the speed of technological change, leaving many workers behind. As inequality has risen, political tensions (P) have emerged between different socio-economic groups. In many countries, the working class has expressed frustration over stagnant wages and job losses, while elites in the tech and finance sectors have thrived. These tensions have manifested in populist movements, protests, and rising polarization in political discourse. In some cases, these movements have challenged traditional political elites, leading to the election of outsider candidates and shifts in policy priorities. Over time, social norms (S) have adapted to these changes, reinforcing existing power structures. In some societies, the narrative of technological progress has been used to justify growing inequality, with arguments that innovation and efficiency will eventually “trickle down” to benefit all. This has led to greater acceptance of income and wealth disparities, and in some cases, has slowed efforts to reduce inequality through stronger redistributive policies. Nevertheless, this also results in lower social mobility, as access to opportunities becomes increasingly tied to one’s position in the socio-economic hierarchy.  Therefore, current policymakers are suggested to be proactive and keep updated in their interventions to counteract inequality arising from new technologies. Progressive tax policies, redistribution mechanisms, and investments in education and reskilling programs could help ensure that the benefits of technological innovation are more evenly distributed. |
| 4. Other minor comments: missing information or typo, such as Page 3 first paragraph, an empty parenthesis. Add short explanations under tables and figures for better readability | Thanks for pointing it out. We have corrected the typo now. |
